# Molecular Formula Identification with SIRIUS

**DOI:** 10.3390/metabo3020506

**Published:** 2013-06-13

**Authors:** Kai Dührkop, Kerstin Scheubert, Sebastian Böcker

**Affiliations:** Lehrstuhl für Bioinformatik, Friedrich-Schiller-Universität Jena, Ernst-Abbe-Platz 2, 07743 Jena, Germany; E-Mails: kai.duehrkop@uni-jena.de (K.D.); kerstin.scheubert@uni-jena.de (K.S.)

**Keywords:** CASMI, mass spectrometry, small molecules, SIRIUS, molecular formula identification, isotope patterns, fragmentation patterns

## Abstract

We present results of the SIRIUS^2^ submission to the 2012 CASMI contest. Only results for Category 1 (molecular formula identification) were submitted. The *SIRIUS* method and the parameters used are briefly described, followed by detailed analysis of the results and a discussion of cases where SIRIUS^2^ was unable to come up with the correct molecular formula. SIRIUS^2^ returns consistently high quality results, with the exception of fragmentation pattern analysis of time-of-flight data. We then discuss possibilities for further improving SIRIUS^2^ in the future.

## 1. Introduction

Structural elucidation and identification of small molecules plays an essential role in many areas of biology and medicine. Mass spectrometry (MS) is a key analytical technique for analyzing these compounds. Compared with nuclear magnetic resonance spectroscopy, MS is orders of magnitude more sensitive. 

In principle, LC-MS/MS enables the identification of small molecules. However, the automated interpretation of such data is still in its infancy. Searches in spectral libraries often fail due to their incompleteness and the varying LC-MS/MS spectra between different instruments [1,2]. Although, at least for some libraries, the variability of fragmentation seems to be a minor problem, it does complicate the search and, especially, the decision of whether or not a spectrum only matches by chance. The development of computational methods for small molecular MS is further impeded by the absence of openly available benchmark test sets. These provide developers of novel approaches to the problem with the required input for training and evaluation of their methods. Recently, a first benchmark dataset for identifying small molecules by GC-MS and LC-MS data was made available for the Critical Assessment of Small Molecule Identification (CASMI) challenge. 

Several promising methods for automated structural elucidation have recently been developed [3,4,5]. All these approaches perform searches in molecular structure databases, which are filtered by the mass of the molecular ion within a given range. Even with this filtering, this subset can be quite large. In order to reduce the search space, and with it, false identifications, it would be useful to determine the molecular formula of the compound before starting a search. Here, we describe how our method, SIRIUS^2^ [6,7], can be used to determine molecular formulas based on isotope pattern analysis and calculation of fragmentation trees. 

Our paper is submitted as part of the Critical Assessment of Small Molecule Identification (CASMI) contest, 2012. More detailed descriptions of the methods have previously been published elsewhere, see [6,8,9,10,11,12]. We focus on the compounds provided by the CASMI challenge, describing in detail the performance of, and issues with, our method. 

Since SIRIUS^2^ can only identify the molecular formula and not the structure of compounds, we restricted ourselves to the first category of the contest. GC/MS challenges were not attempted because they have only unit mass resolution. The first category consists of 14 challenges: six compounds measured on a TOF instrument (Bruker micrOTOF-Q) and eight compounds measured on a Thermo Orbitrap instrument. Each measurement consists of MS and MS^2^ spectra. 

## 2. Methods

SIRIUS^2^ analyzes the isotope pattern in MS spectra, together with the fragmentation pattern of MS^2^ spectra, to determine the molecular formula of the measured compound. Isotope pattern analysis is described in detail in [6]. We first decompose the monoisotopic peak and filter the list of candidate formulas using Senior’s rule [13]. Unless specified otherwise, we use the most common naturally occurring elements carbon (C), hydrogen (H), nitrogen (N), oxygen (O), phosphorus (P) and sulfur (S). Without any knowledge of the ionization of the compound, it is not possible to restrict our search to formulas with integer ring double bond equivalent (RDBE) values. We filter out all candidates with RDBE values lower than −0.5. For the remaining decompositions, we compute a theoretical isotope pattern by convoluting isotope distributions. Again, when not stated otherwise, we assume that the measured compounds contain naturally distributed isotopes. We then compare the theoretical isotope pattern with the measured one and compute a likelihood. See Section 2.1 below for details. 

MS^2^ spectra are analyzed using fragmentation trees [10], see [12] for the evaluation protocol. We merge MS^2^ spectra and decompose all fragment peaks. For each decomposition of the parent peak, we build a fragmentation graph, which contains all possible explanations for each peak, as well as all possible fragmentation reactions between the peak explanations. We weight the edges, using a scoring scheme loosely based on the logarithmized likelihood that a certain fragmentation reaction occurs. We then compute a subtree of maximum weight. This tree decides, implicitly for each peak, whether it is noise, and if not assigns it the molecular formula of the corresponding fragment, as well as the fragmentation cascades. The score of the tree is the sum of its edge weights. See Section 2.2 for details. 

For each candidate, we combine both scores by multiplying the isotope pattern score by a factor of 5 and adding it to the fragmentation tree score. This weighting reflects the higher reliability of isotope patterns compared with fragmentation patterns. Molecular formulas are then ranked by combined score. Finally, we try to determine the molecular formula of the neutral compound by testing different ionizations and searching for the molecular formula of the molecule in PubChem. 

### 2.1. Isotope Pattern Analysis

To score the similarity between measured spectra and theoretical isotope patterns, we calculate likelihoods and posterior probabilities using Bayesian Statistics. The fundamentals of this approach have been described in [6]. Matching of peaks between a theoretical pattern and a measured pattern, for the given data, is a trivial task, as we have usually only one peak per nominal mass. If we have more than one peak per nominal mass, we select the peak with the highest intensity and assume the others to be noise. Special cases, such as peaks that have the same nominal mass but stem from different isotope species or overlapping isotope patterns, do not appear in the challenge data. Peaks with relative intensities measured below 2% are omitted. For each matched pair of measured and theoretical isotope peaks, we calculate likelihoods from deviation in mass and intensity. 

**Figure 1:**
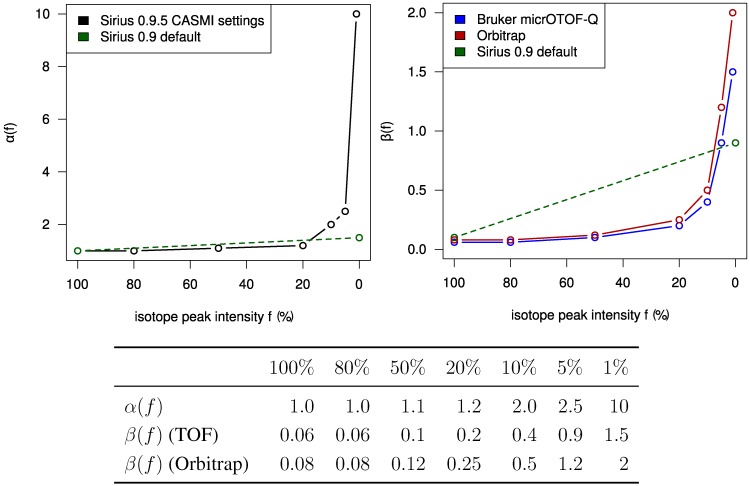
Parameter values depending on the peak intensity. In SIRIUS^2^ 0.9, *α*(·) and *β*(·) are linear functions (green dashed lines). In SIRIUS^2^ 0.9.5, these are replaced by piecewise linear functions. Left: Mass accuracy factor *α* for varying peak intensity; Right: Intensity accuracy *β* for varying peak intensity. We choose a higher intensity accuracy for TOF than for Orbitrap. Bottom: Interpolation points for linear interpolation of *α*(·) and *β*(·).

Mass deviations are assumed to be normally distributed and standard deviation to increase with decreasing intensity. The logic behind this assumption is that accurate peak picking becomes increasingly tedious for low intensity peaks. Assuming that more than 99.97% of peaks measured have a lower deviation in mass than given mass accuracy (measured as parts per million, ppm) we calculate the standard deviation by

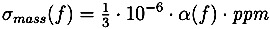

where *f* is the intensity of the measured peak. For Orbitrap data we assume mass accuracy to be *ppm* = 5, for TOF data we distinguish between positive spectra with accuracy *ppm* =7 and negative spectra with *ppm* = 10. The function *α* is chosen such that peaks with lower intensity have higher mass deviations (Figure 1). Unlike [6], we do not use a linear function for this purpose, as this overestimates the problem for relative intensities of 20% but underestimates it for those of 2%. Instead, we use a piecewise linear function (linear interpolation), see Figure 1. The probability of observing the mass deviation between the measured peak *M* and the theoretical peak *m* is calculated as 

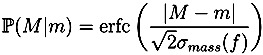

For all but the monoisotopic peaks, we subtract the monoisotopic mass from the peak masses using mass differences to avoid penalizing calibration errors (or any other bias) multiple times. 

For intensities, we assume that the logarithmized ratio between measured and theoretical intensity is normally distributed. Peak intensities are normalized such that they sum to 100% and log-ratios between the measured and theoretical intensities are then computed. We define the standard deviation of this ratio depending on the intensity of the peak: 

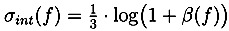

where *β* increases with decreasing intensity, again using a piecewise linear function (Figure 1). Finally, a bias in the intensities of Orbitrap data was detected, which leads to an underestimation of the monoisotopic peak intensity [6]. We compensate for this by adding an offset of 0.2% to the peak intensities of Orbitrap spectra before renormalizing them. The probability of observing a deviation in intensity between a measured intensity *f* and a theoretical intensity *p* is calculated as

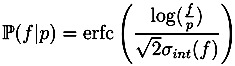


All parameters for *α*(·), *β*(·) and the peak intensity offset compensation were not optimized for the present data but rather chosen *ad hoc* by educated guesses. The isotope pattern score is the sum of the logarithmized probabilities of each peak. 

### 2.2. Fragmentation Pattern Analysis

We follow the evaluation protocol described in [12]. For each candidate formula a fragmentation graph is built, containing vertices for all possible explanations per peak (each molecular formula within the mass accuracy of the instrument). We draw directed edges (arcs) between vertices if the molecular formula of the latter vertex is a sub-formula of the first one. Vertices are scored as log-odds of the likelihood of observing such a mass deviation and the likelihood that the peak is noise. Again, the mass deviations are modelled according to a normal distribution. For fragments we assume a maximal mass deviation of 10 ppm. The noise probability is modelled as an exponential distribution with parameter λ = 16. A heteroatom-to-carbon distribution, gleaned from the KEGG COMPOUND database [14], adds prior probabilities to the vertices to score formulas according to their “chemical reasonability”. Further, we penalize fragmentation reactions where the parent fragment occurs at a higher collision energy than the child fragment. The edge scores are modified using the list of common losses and implausible losses from [12]. In addition, edges are penalized by losses that have a high mass, relative to the mass of the corresponding fragments. After weighting the edges and vertices, we search for the subtree of maximum weight, which explains each peak by at most one molecular formula and which assigns a unique fragmentation reaction for the generation of each fragment peak [15]. 

### 2.3. Data Processing

Rather than picking peaks from raw data, peak lists provided by CASMI were used. This analysis was done using the SIRIUS^2^ 0.9.5 command line tool (The command line tool SIRIUS^2^ version 0.9.5 will be made available at [16]). The molecular formula identification of SIRIUS^2^ operates without user interaction, other than assigning of parameters (ppm, *α* and *β*). Different parameters for TOF and Orbitrap data as well as positive and negative mode spectra were selected. For the two negative mode spectra, we used a relative intensity threshold of 0.5%, as these spectra appear to contain an exceptionally high number of noise peaks. For Challenges 11, 12 and 13 more than one compound was found in the MS spectrum. Here, we repeated the analysis for each separate isotope pattern and chose the compound and molecular formula whose isotope pattern scored the highest. 

The compound in Challenge 16 undergoes in-source fragmentation. That is, the MS spectrum potentially does not contain the parent peak but fragment peaks and isotopes of them. We merged the fragmentation patterns, manually inserted the compound mass of *m*/*z* 359.1481 from the challenge description and computed fragmentation trees, which include the isotope scores of the fragments at *m*/*z* 150 and *m*/*z* 170, into the scoring of its vertices. 

The results of our software are logarithmized probability values, together with the formulas of the ion. Because CASMI allows only positive scores and neutral formulas, we had to do some post processing: Positive scores were calculated by taking the exponential of the log probabilities. For the ionization of positive mode spectra, we had to distinguish between protonated compounds and intrinsically charged molecules. Other ionizations such as [M + Na]^+^ were also tested but these did not result in top scores and were omitted from further analysis. As we were unsure of how to directly derive the ionization from the data (using, say, the RDBE value) we instead relied solely on PubChem to distinguish these two cases. For the protonation hypothesis a single hydrogen was extracted from the TOP 5 molecular formulas and these formulas were used to search in PubChem. In cases where a hit is found, the search stops and protonation assumed. If no hit is found, we search with the molecular formula of the ion itself in PubChem. Due to the relative rarity of intrinsically charged molecules, the protonated version of the compound is also added, with a lower score, to our output list. When neither the protonated nor the ionic molecular formula can be found in PubChem, we return the molecular formula, minus a single hydrogen and assume that the compound is not currently stored in PubChem. For negative mode spectra, we distinguish between deprotonated and intrinsically charged molecules. 

Other information from PubChem such as chemical or biological reasonability of the compound was not used. On application of the software, this could be performed as post-processing, but SIRIUS^2^ provides a pure, *de-novo* approach for analyzing mass spectra. 

## 3. Results and Discussion

The first category of the contest consists of 14 challenges: six compounds measured on a TOF instrument and eight compounds measured with an Orbitrap instrument. Four of the TOF measurements had much higher mass deviations than stated in the contest description. These compounds were unidentifiable by SIRIUS^2^ for obvious reasons. After the contest, new, recalibrated spectra were uploaded by the CASMI organizers. We repeated the analysis, with the same parameters, on this data. For Challenge 5 the data initially provided proved to be more accurate than the recalibrated data. The recalibrated spectra showed a higher mass deviation and the results were therefore of less use than the initial spectra. For the other compounds, our results improved greatly and often allow identification of the correct molecular formula solely from the isotope pattern data. 

For the recalibrated TOF spectra we scored four out of the six compounds with rank 2. In fact, we found that for these compounds we were able to identify the correct molecular formula using only the isotope pattern data due to high isotope pattern accuracy. By contrast, the best rank of any correct molecular formula in the fragmentation pattern data analysis is 7. This gives combined scores with worse identification levels than the pure isotope pattern analysis. For the Orbitrap data, we identified 6 of 8 compounds with rank 1. One of these compounds was predicted with the wrong number of hydrogens. Here, we see that both the isotope pattern analysis and the fragmentation pattern analysis provide good identification results. All in all, our analysis identified the correct molecular formula for 10 out of the 14 compounds with a rank of 1 or 2. Using solely the isotope pattern data, the molecular formulas of these 10 compounds were correctly identified as the top rank. See Table 1 for details. In contrast to these results, we have shown in a previous study that fragmentation pattern analysis of SIRIUS^2^ improves molecular formula identification [11]. Here, 28 of the 37 compounds were ranked at 1 by solely using isotope pattern analysis, but eight compounds were only ranked top by taking into account additional information from fragmentation trees. The relatively large errors in some of the fragmentation spectra have complicated the calculation of good fragmentation trees. However, using the example of Challenge 3 for which the combined rank outperforms the two single ranks, we can see the potential of fragmentation trees for molecular formula identification. 

A possible explanation for the poor performance of SIRIUS^2^ on TOF fragmentation data is the relatively poor mass accuracy of this data: In our analysis we assumed a mass accuracy of 10 ppm, which implies that 99.7% of the measured fragment peaks fall within this mass deviation. However, as we also assume a normal distribution of mass deviation, this implies that 68.2% of the peak mass deviations are less than 3.33 ppm. When using the correct molecular formula as the root, the fragmentation trees computed by our approach show much higher mass deviations. Possibly, the mass accuracy of the TOF measurements is insufficient for fragmentation tree analysis. This idea was further supported by manual analysis of the fragmentation trees computed with correct molecular formulas, which showed somewhat strange “topological features”. 

**Table 1:** Details of the challenge compounds and rank of the correct molecular formula for isotope pattern data, fragmentation pattern data and a combination of both scores. TOF data (1–6) and Orbitrap data (10–17). For Challenges 2, 4, 6 we used the recalibrated data. For Challenge 5 we used the *original* data, as mass accuracy after recalibration appears to be even worse.

Chal. No.	molecular formula	parent peak (m/z)	mode	No. of isotopes	rank isot. pattern	rank frag. pattern	combined rank
1	C_18_H_36_N_4_O_11_	485.245	positive	3	15	7	**3**
2	C_28_H_32_O_14_	591.171	negative	4	**1**	604	2
3	C_14_H_27_NO_9_S_3_	448.075	negative	5	**8**	12	**8**
4	C_19_H_17_NO_4_	324.122	positive	3	**1**	8	2
5	C_19_H_23_NO_4_	330.171	positive	4	**1**	18	2
6	C_21_H_21_NO_6_	384.144	positive	4	**1**	15	2
10	C_14_H_9_NO_2_	224.071	APCI positive	3	**1**	5	**1**
11	C_17_H_12_O	231.080 *	APCI positive	2 *	1 *	5 *	1 *
12	C_17_H_16_N_4_O_4_	341.126	APCI positive	2	**13**	21	18
13	C_19_H_17_OP	293.110	ESI positive	3	**1**	2	**1**
14	C_12_H_9_N	168.080	APCI positive	2	**1**	**1**	**1**
15	C_12_H_13_NO_2_	204.102	APCI positive	2	**1**	**1**	**1**
16	C_18_H_21_N_3_O_5_	-	APCI positive	-	N/A	5 (2 **)	5 (2 **)
17	C_13_H_13_N_3_	212.119	ESI positive	3	**1**	2	**1**

* For Challenge 11 we wrongly interpreted the parent peak and its fragment as isotope pattern. We reported a molecular formula of C_17_H_11_O which differs only by a single hydrogen from the correct molecular formula of C_17_H_12_O; ** Due to an operating error we used the wrong parent mass in our initial analysis; the number in brackets is the rank when using the correct parent mass.

The CASMI contest provides spectra from different instruments, ionization modes and shows several pitfalls; but for each of these categories, the number of spectra is very small. Although this is a good way to evaluate the flexibility of the competing tools, using only the number of correct identifications complicates the interpretation of results. To this end, we discuss the complications and problems we have run into. 

For Challenge 1, it appears that the mass accuracy for both the isotope pattern and fragmentation data was simply not sufficient for our analysis. Given this low mass accuracy, the possible explanations for the fragments and hence the size of the fragmentation graph explode. In such a graph, incorrect common losses can be inserted purely by chance. This decreases the discriminating power between the correct and incorrect trees and thus complicates identification of the correct molecular formula. 

For Challenge 2 the fragmentation pattern analysis completely fails. The correct fragmentation tree is ranked in 604. We found that the mass deviation of the fragments is too high for reasonable analysis. 

For Challenge 3 the correct answer was assigned rank 8. This was due to the low quality of the isotope pattern. The fragmentation tree from fragmentation pattern analysis was empty. A closer look into the data shows that the intensities of the MS^2^ spectra are dominated by the parent peak. We assumed that the peaks at *m*/*z* 96 and *m*/*z* 97 were noise peaks as no molecular formulas matched the masses within 10 mDa. The remaining peaks have very low relative intensities, below 2%. Because SIRIUS^2^ uses relative intensities normalized over the merged spectra in its scoring, all peaks, apart from the parent peak, are labelled as noise. Setting the intensity of the parent peak to zero increases the relative intensities of the other peaks. Most of the fragment peaks are now annotated, resulting in the correct identification of the compound at rank 1. Challenge 3 exposes a shortcoming in the fragmentation pattern analysis of SIRIUS^2^: It fails if the intensity of the parent peak is too large in comparison with the fragment peaks. A simple solution for this problem is to normalize the spectra without the parent peak. 

The molecular formula for Challenge 11 was not found by SIRIUS^2^. Here it suggested the molecular formula C_17_H_11_O, whereas the correct answer is C_17_H_12_O. Because the challenge description contains no information about the parent peak, we mistakenly assumed the peak at *m*/*z* 231.0798 to be the parent peak and interpreted the real parent peak, at *m*/*z* 232.0831, as an isotope peak. This assumption seemed to be reasonable as the resulting isotope pattern is very accurate. 

In Challenge 12, we chose the wrong parent peak. We assumed that the MS/MS spectrum belongs to the peak at *m*/*z* 363.1079. With this as parent peak, we predicted a compound with formula C_18_H_18_O_8_. The correct parent peak is at *m*/*z* 341.1260 with formula C_17_H_16_N_4_O_4_. The peak at *m*/*z* 363.1079 seems to be a sodium adduct of the compound. In principle, our algorithm can handle different adducts, but it must be explicitly stated which fragmentation spectrum belongs to which parent peak. After calibrated spectra were uploaded, the correct parent mass was added to the challenge description. Unfortunately, SIRIUS^2^ ranked the correct molecular formula at rank 18. We found that practically all peaks show a mass bias of about 6 ppm. 

In Challenge 16 the parent peak was not contained in the MS spectrum due to strong in-source fragmentation. The neutral mass of the parent peak was, however, provided in the challenge description. Here, we incorrectly used the neutral mass instead of the ionized mass as input. This is not an issue of SIRIUS^2^ but rather an operating error. The correct compound was again predicted at rank 5 with the wrong number of hydrogen atoms. SIRIUS^2^ expects ionized masses and subtracts an H^+^ from the input mass. This lead to an erroneous number of hydrogens and, further, to a shift in the mass difference between hydrogen and proton. If we redo the analysis with the correct mass we get the correct molecular formula at rank 2. 

**Figure 2:**
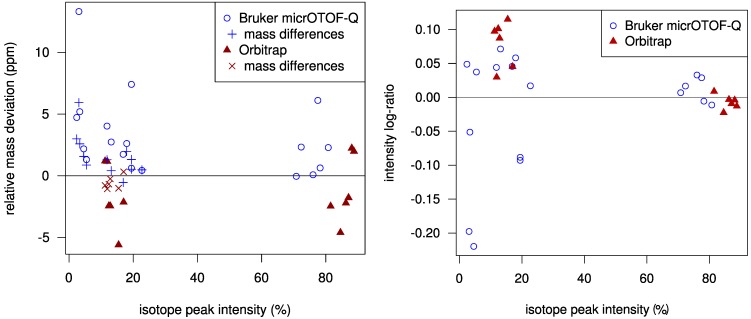
Left: Relative mass deviation plotted against relative intensities of isotope peaks. For all but the monoisotopic peak, we use the mass deviation of the difference between the peak mass and the monoisotopic peak mass; Right: Logarithmic ratio of measured to theoretical isotopic peak intensity (log-ratio) plotted against relative peak intensities. For Orbitrap data, the log-ratio of the monoisotopic peaks is mostly negative and is always positive for the +1 peaks. The variance of the log-ratios is higher for lower intensities.

Using a statistical model for the scoring allows us to check how well the challenge data fits our model. In Figure 2 we plot the mass deviations of the isotope peaks from the simulated isotope patterns. We find that deviations in mass differences of isotope peaks are usually smaller than deviations in the monoisotopic peak mass. We evaluate the intensity deviations analogously, see again Figure 2. For most Orbitrap spectra, our intensity offset of 0.2% was reasonable. In contrast, for the TOF spectra we correctly decided to set this offset to zero. Possibly, this offset is a bias typical for Orbitrap measurements. Finally, it appears that we slightly underestimated the intensity accuracy for both instruments. 

## 4. Conclusions

SIRIUS^2^ is a software tool for automatically determining the molecular formula of compounds from their mass spectral data alone. Combining isotope and fragmentation pattern analysis, it requires no user interaction, and no compound databases or spectral libraries are accessed. SIRIUS^2^ identified the chemical formulas of the majority of the compounds measured on an Orbitrap instrument, whereas, for TOF measurements, the correct molecular formula was within the first two suggestions in most cases. We have highlighted some pitfalls and how they might be avoided in the future. “Side-products” of the SIRIUS^2^ analysis are fragmentation trees. These can be used for further classification and clustering of the compounds [12]. 

The failed challenges indicate a potential direction for improvements of SIRIUS^2^: We require an automated approach for detecting such errors. In addition, recalibration can help to improve identification results: Experimental results show that a recalibration based on uniquely decomposable peaks improves performance significantly [17]. Our evaluation also indicates that we should use different mass deviation functions for monoisotopic peak masses and the more accurate peak mass differences. 

Many challenges in the CASMI contest deal with atmospheric pressure chemical ionization (APCI). SIRIUS^2^, however, was developed for and evaluated with electrospray ionization (ESI) in combination with collision-induced dissociation (CID) tandem MS data but was not developed for phenomena such as in-source fragmentation. In Challenge 16, for example, we had to write a manual workaround to handle this. Hufsky *et al.* [18] have developed a method for analyzing Electron Ionization (EI) fragmentation data, which could possibly be adapted for in-source fragmentation data. 

SIRIUS^2^ outputs a candidate list with scores based on posterior probabilities. These depend heavily on the chosen model and parameters. At the moment, our results should be interpreted as a way of ranking the potential molecular formula, but cannot be used as an estimate whether the answer is correct. 

Generally, the number of spectra and compounds analyzed is much higher than the 14 compounds from CASMI. For large datasets SIRIUS^2^ may perform better if its parameters are trained on reference measurements. Despite this, the current challenge setup favors a semi-automated analysis. For future challenges, we propose using a different setup: A batch of compound spectra (around 100) measured on a single instrument should be given to the contestants to train the method’s parameters. Then, another batch of spectra (possibly another 100) measured on the same instrument should be analyzed in a fully automated manner, using the parameters from the first batch. 
